# 15q23 Gain in a Neonate with a Giant Omphalocele and Multiple Co-Occurring Anomalies

**DOI:** 10.1155/2018/8702568

**Published:** 2018-11-13

**Authors:** Hui-Fang Zhou, Christopher J. O'Conor, Chiraag Gangahar, Louis P. Dehner

**Affiliations:** ^1^Barnes-Jewish Hospital, Saint Louis, MO 63110, USA; ^2^The Department of Pathology and Immunology, Washington University School of Medicine, Saint Louis, MO 63110, USA

## Abstract

**Background:**

Omphalocele is a rare congenital abdominal wall defect. It is frequently associated with genetic abnormality and other congenital anomalies, although isolated omphalocele cases do exist. Data have shown that omphalocele with co-occurring genetic abnormality has worse prognosis than isolated omphalocele. Chromosomal analysis by a conventional technique such as karyotyping can only detect aneuploidy and large segmental duplication or deletion. Newer techniques such as high-resolution microarray analysis allow for the study of alterations in chromosomal segments that are less than 5 Mb in length; this has led to identification of critical region and genes in the pathogenesis of omphalocele.

**Case Presentation:**

The current study is the initial report of a newborn male with a 15q23 gain and a giant omphalocele. High-resolution chromosomal microarray analysis identified this gain of copy number spanned 676 kb, involving almost the entire *NOX5* gene (except for exon 1 of the longer transcript), the entirety of the *EWSAT1*, *GLCE*, *PAQR5*, *KIF23*, *RPLP1*, and *DRAIC* genes and exons 1–3 of the *PCAT29* gene.

**Conclusion:**

To date, this is the first report of an associated 15q23 gain in a case with omphalocele. Interestingly, Giancarlo Ghiselli and Steven A Farber have reported that GLCE knockdown impairs abdominal wall closure in zebrafish. We also identified GLCE gene alteration in our case. This highlights the importance of GLCE in abdominal wall development. Further study of the function of GLCE and other genes might lead to a better understanding of the molecular mechanism of omphalocele.

## 1. Background

An omphalocele is a rare congenital abdominal wall defect of the umbilical ring. The prevalence in the United States during the period 1995–2005 was 1.92 per 10,000 live births with a slight male preference [1]. The defect is characterized by eviscerated abdominal contents covered by a 3-layer membrane: amnion externally, peritoneum internally, and mesenchyme or Wharton's jelly between the two former layers [2]. Omphalocele has been classified on the basis of size (minor or giant), the integrity of the sac (intact or ruptured), contents (liver-containing or absent), and with or without concurrent anomalies [3].

Giant omphaloceles have been defined by various criteria, including the diameter of the sac or abdominal wall defect, ability or inability for a primary closure at the defect, a tissue defect >5 cm, liver-containing herniation of viscera, and volume disproportion between the abdominal viscera and abdominal cavity. Clinically, an omphalocele containing the liver of at least 4.5–5 cm in diameter is defined as a giant omphalocele [2].

Risk factors for omphalocele include young (<20 years old) or advanced maternal age (>35 years old), multiparity, prenatal alcohol exposure, maternal smoking, pre-pregnancy overweight or obesity status, maternal asthma medication, maternal intake of selective serotonin reuptake inhibitors, male neonates, and multiple births. Omphalocele has a high co-occurrence with other musculoskeletal, digestive, genitourinary, and cardiovascular system-related anomalies, chromosomal anomalies, and nonchromosomal syndromes. Chromosomal abnormalities, including full and partial aneuploidies, have been reported in 15%–20% of the neonates and 30% of the fetuses with omphaloceles; this disparity is due to the high rate of pregnancy terminations (30–50%) and spontaneous abortion [4].

We report a newborn with a giant omphalocele whose chromosomal microarray revealed a chromosome 15q23 gain, spanning approximately 676 kb in length.

## 2. Case Presentation

A newborn boy was born at 39 weeks gestational age (GA) by a scheduled cesarean section with a birth weight of 2480 g. The mother is a 41-year-old gravida 2 with a term, vaginal delivery in 1996, which was complicated by meconium aspiration requiring neonatal intensive-care unit admission. The maternal history is complicated by morbid obesity with a body mass index (BMI) over 50, untreated hypothyroidism, long-standing history of asthma, and bipolar disorder. Medications included hydroxyzine for anxiety attacks. The mother denied alcohol, tobacco, and illicit drug use during the pregnancy.

The pregnancy was unexpected and was discovered during an emergent department visit at 27 weeks GA. A prenatal ultrasound performed at 32 weeks GA showed multiple fetal anomalies including an omphalocele, lumbar spine angulation, narrowed thoracic cage, and 2-vessel, short umbilical cord. A fetal MRI at 34 weeks GA confirmed the previous ultrasonographic findings, with the addition of a hypoplastic left lung (23% of expected lung volume of GA), and high-riding left kidney. The family was consulted as to the severity of the various abnormalities and the options. However, due to the family's religious belief, a termination of the pregnancy was not considered. The decision was made for a term cesarean section delivery and comfort care after the baby was born. The baby was born without significant respiratory effort, an APGARS score of 1 at 1 minute and 5 minutes. The baby died 95 minutes after birth.

## 3. Pathologic Findings

**Figure 1:**
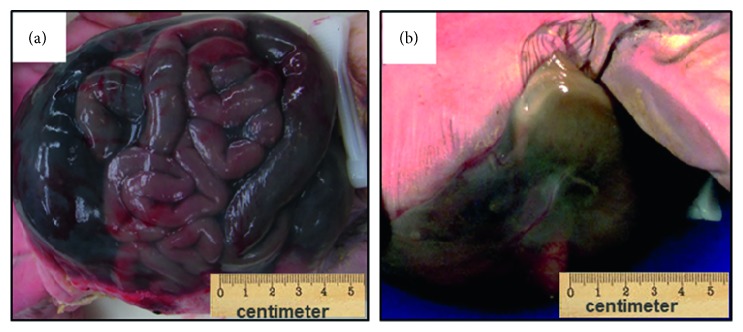
The infant has a giant liver containing omphalocele. (a) The omphalocele is covered with an intact, translucent membrane. The liver, spleen, stomach, and malrotated small and large intestine are present in the sac. (b) The omphalocele is associated with an 8 cm middle abdominal defect.

**Figure 2:**
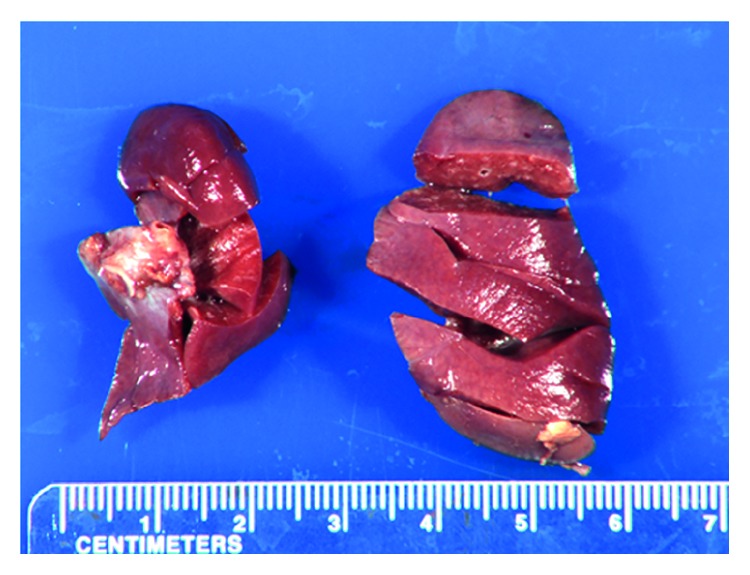
The lungs are hypoplastic. The left and right lung has the normal lobe configuration. The combined weight of the lungs is below 5 percentile of the lung weight of the GA.

The omphalocele containing the liver, spleen, stomach, and malrotated small and large intestine had a height of 12.5 cm and an intact, translucent sac (Figure 1). The abdominal wall defect measured 8.0 cm in diameter (Figure 1). The narrowed chest circumference was 23.4 cm (normal reference range: 30.5–33 cm). The combined lung weight was 9.3 g (left: 3.7 g, right: 5.6 g), which is less than 5 percentile of the normal lung weight for the GA (Figure 2). The posterior diaphragm was contiguous with the omphalocele sac with cardiac herniation. The left kidney was displaced upward and compressed the left lung. The lumbar spine was angulated (a fetal MRI at 34 weeks GA showed a lumbar spine angulation approximately 90°). The umbilical cord measured 5.6 cm by length and 1.0 cm by diameter and contained a single artery and a vein. The heart and the great vessels are inspected very carefully both macroscopically and microscopically. There was no congenital cardiac abnormality noted on the baby. The sections of the left and right ventricle have no histopathologic abnormality.

## 4. Genetic Studies

**Figure 3:**
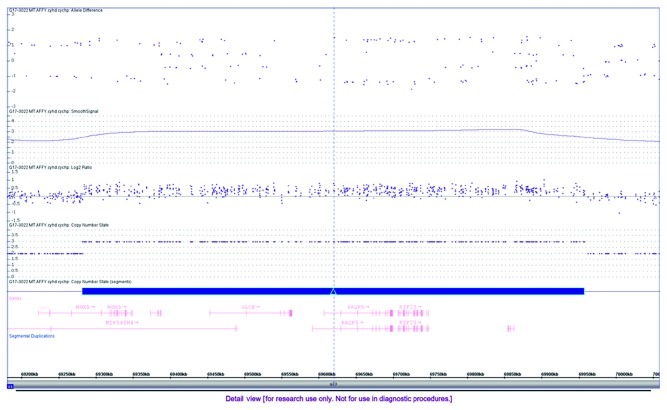
Chromosomal microarray shows that the neonate carries 15q23 duplication, spanning approximately 676 kb in length and consisting of 660 markers/probes.

Skin fibroblasts were cultured, and chromosomal microarray analysis was performed to assess copy number gains/losses using the Affymetrix CytoScan HD array. The genotype was 46, XY with 15q23 gain of copy numbers. Chromosomal microarray analysis revealed an interstitial gain on the long arm of chromosome 15 at q23, spanning approximately 676 Kb in length and consisting of 660 probes (Figure 3). The copy number gain at 15q23 involved almost the entirety of the *NOX5* gene (except for exon 1 of the longer transcript), the entirety of the *EWSAT1*, *GLCE*, *PAQR5*, *KIF23*, *RPLP1*, and *DRAIC* genes and exons 1–3 of the *PCAT29* gene. The maternal and paternal genetic study revealed that the newborn's genetic mutation is inherited from the maternal chromosome, while the paternal chromosome contains a normal sequence at 15q23 region.

## 5. Discussion

We report a case of a neonatal male with a giant omphalocele, associated with a narrowed thorax and severe pulmonary hypoplasia, diaphragmatic defect with cardiac herniation, lumbar spine angulation, intestinal malrotation, and a short, two-vessel umbilical cord. High-resolution chromosomal microarray analysis identified a 15q23 gain spanning 676 kb, involving almost the entire *NOX5* gene (except for exon 1 of the longer transcript), the entirety of the *EWSAT1*, *GLCE*, *PAQR5*, *KIF23*, *RPLP1*, and *DRAIC* genes and exons 1–3 of the *PCAT29* gene. No similar gains have been described in the DGV, DECIPHER, and ClinVar databases, although larger duplications overlapping this region have been identified in infants with omphalocele and concurrent other anomalies.

Omphaloceles are frequently associated with other anomalies as in our case. A study of 2,308 clinically confirmed omphaloceles over 11 years from 12 state population-based birth defects registries showed that approximately 20% of omphaloceles occurred without other birth defects [1]. Although patients with isolated minor and giant omphalocele might be managed by different surgical strategies, they have similar long-term prognosis and comparable life quality after a high level of medical intervention in early life [3]. A recent study of isolated minor and giant omphaloceles revealed that approximately 17% neonates with an omphalocele also had chromosomal anomalies, 32% had congenital heart defects, 8% had central nervous system defects, and the remaining 22% had defects that spanning every organ system. Interestingly, multiple associated anomalies appear to be more common with a minor omphalocele than a giant omphalocele (55% vs 36%) [5, 6].

The most common chromosomal abnormality associated with an omphalocele is trisomy 18 (50.1%) [7], trisomy 13 (28.8%) [8], and trisomy 21 (8.3%) [1, 9]. Other less common chromosomal abnormalities reported co-occurring with an omphalocele include triploidy, 45, XO, 47, XXY, and 47, XXX [4]. A wide range of partial aneuploidy, including dup (3q), dup (11p) [10], inv (11), dup (1q), del (1q), dup (4q), dup (5p), dup (6q), del (9p), dup (15q) [11], dup (17q), dup (7q)/del (21q) [12], dup (3q)/del (9q) [13], Pallister-Killian syndrome with mosaic tetrasomy 12p and Miller-Dieker lissencephaly syndrome with deletion of 17p13.3, and uniparental disomy (UPD) such as UPD11 and UPD14, is also reported to be associated with omphalocele [4, 10, 11, 14, 15].

Lacro and associates reported a compound chromosome 15 long arm terminal duplication and chromosome 13 long arm terminal deletion (15q22 ⟶ qter/13q32.3 ⟶ qter) in an aborted fetus who had an omphalocele and a cephalic defect in neural tube closure [11]. Our case of giant omphalocele with multiple co-occurring congenital anomalies had a 15q23 gain which highlights the seeming importance of 15q23, specifically *NOX5*, *EWSAT1*, *GLCE*, *PAQR5*, *KIF23*, *RPLP1*, *DRAIC*, and *PCAT29* gene, in the embryogenesis and development of the abdominal wall. Interestingly, Giancarlo Ghiselli and Steven A Farber have reported that GLCE knockdown impairs abdominal wall closure in zebrafish [16]. We also identified GLCE gene alteration in our case. These data highlight the importance of GLCE in abdominal wall development. Further study of the function of GLCE and other genes might lead to a better understanding of the molecular mechanism of omphalocele.

The formation of small and giant omphaloceles seems to involve different mechanism [17]. The mechanism of the formation of small omphaloceles is relatively straightforward. The midguts begin to grow rapidly during the 6th week of gestation, resulting in the normal herniation of the intestines through the umbilical ring and the midgut begins to rotate and return to the abdominal compartment by the 10th week GA. If the intestine fails to return to the abdominal compartment, a small hernia into the umbilicus occurs, resulting in a small omphalocele, with minimal widening of the umbilical ring [18]. However, the embryological events associated with large omphaloceles are more complicated and less settled in terms of pathogenesis. It is proposed that the formation of epigastric, hypogastric, and midabdominal wall defects may involve different events [6]. Wilson and colleagues proposed that an abnormality in body folding in the cephalic region of the fetus results in an epigastric omphalocele, while a caudal folding abnormality causes a low or hypogastric omphalocele often in association with bladder or cloacal exstrophy, and anomalous lateral body wall folding results in midabdominal defects [2]. The process of fusion likely involves the combination of cell proliferation, cell migration, production of extracellular matrix, apoptosis, and specialized cell-to-cell contacts. Several genes and cell signaling pathway have been implicated in the process. *Msx1* and *Msx2* double-mutant mice have the omphalocele phenotype with disorganized abdominal muscle layers and connective tissues [19]. Doi and colleagues reported that the administration of Cadmium during the critical period of embryogenesis induced an omphalocele phenotype in the chick embryo; the mechanism relates to the downregulation of *Msx1* and *Msx2* expression [17]. Another study from the same group demonstrated that cadmium interferes with sonic hedgehog (SHH) signaling pathway by downregulating two critical cell membrane receptors: patched (PTCH) and smoothened (SMO); these studies indicate that *Msx1/Msx2* genes and SHH signaling pathway play an important role during embryonic ventral body wall formation [17]. Our report seemingly highlights the importance of 15q23, specifically *NOX5*, *EWSAT1*, *GLCE*, *PAQR5*, *KIF23*, *RPLP1*, *DRAIC*, and *PCAT29* gene, in the early embryogenesis and abdominal wall development. However, the mechanism by which these genes may regulate the expression of other genes and signaling pathways, leading to the phenotype, remains unknown. Further studies of omphalocele cases with genetic abnormality involving the same region and RNA sequencing and proteomic analysis could lead to a better understanding of the pathogenetic mechanism.

## 6. Conclusion

As our best knowledge, this is the first report of an associated 15q23 gain in a patient with omphalocele. PubMed search with the combination of key words “congenital abdominal wall defect” or “omphalocele” with “genetic” or “chromosomal” returned without any results. This finding is significant in both prognosis of patients with the same genetic abnormality and study of pathogenesis of omphalocele. We believe that future reports of omphalocele patients with genetic abnormality involving the same region and the implementation of RNA sequencing and analysis of their proteomic expression could lead to better understanding of the pathogenesis of omphalocele.
